# Cyclical changes in seroprevalence of leptospirosis in California sea lions: endemic and epidemic disease in one host species?

**DOI:** 10.1186/1471-2334-7-125

**Published:** 2007-11-06

**Authors:** James O Lloyd-Smith, Denise J Greig, Sharon Hietala, George S Ghneim, Lauren Palmer, Judy St Leger, Bryan T Grenfell, Frances MD Gulland

**Affiliations:** 1 Center for Infectious Disease Dynamics, Pennsylvania State University, University Park, USA; 2 The Marine Mammal Center, Marin Headlands, 1065 Fort Cronkhite, Sausalito, USA; 3 California Animal Health and Food Safety Laboratory, University of California, Davis, USA; 4 Research Triangle Institute International, Research Triangle Park, USA; 5 Marine Mammal Care Center, Fort Macarthur, San Pedro, USA; 6 SeaWorld of California, San Diego, USA

## Abstract

**Background:**

Leptospirosis is a zoonotic disease infecting a broad range of mammalian hosts, and is re-emerging globally. California sea lions (*Zalophus californianus*) have experienced recurrent outbreaks of leptospirosis since 1970, but it is unknown whether the pathogen persists in the sea lion population or is introduced repeatedly from external reservoirs.

**Methods:**

We analyzed serum samples collected over an 11-year period from 1344 California sea lions that stranded alive on the California coast, using the microscopic agglutination test (MAT) for antibodies to *Leptospira interrogans *serovar Pomona. We evaluated seroprevalence among yearlings as a measure of incidence in the population, and characterized antibody persistence times based on temporal changes in the distribution of titer scores. We conducted multinomial logistic regression to determine individual risk factors for seropositivity with high and low titers.

**Results:**

The serosurvey revealed cyclical patterns in seroprevalence to *L. interrogans *serovar Pomona, with 4–5 year periodicity and peak seroprevalence above 50%. Seroprevalence in yearling sea lions was an accurate index of exposure among all age classses, and indicated on-going exposure to leptospires in non-outbreak years. Analysis of titer decay rates showed that some individuals probably maintain high titers for more than a year following exposure.

**Conclusion:**

This study presents results of an unprecedented long-term serosurveillance program in marine mammals. Our results suggest that leptospirosis is endemic in California sea lions, but also causes periodic epidemics of acute disease. The findings call into question the classical dichotomy between maintenance hosts of leptospirosis, which experience chronic but largely asymptomatic infections, and accidental hosts, which suffer acute illness or death as a result of disease spillover from reservoir species.

## Background

Leptospirosis is an acute febrile zoonosis of global importance, caused by spirochetes of the genus *Leptospira *[1-5]. Owing to recent epidemics in humans in Nicaragua [6], Brazil [7], India [8] and South East Asia [9], and rising incidence in domestic dogs [10,11], leptospirosis has been identified as an emerging infectious disease [4,12]. Outbreaks in humans are usually seasonal and associated with flooding or activities involving exposure to contaminated water or animal tissues, such as swimming, hunting, farming, and working in abattoirs or veterinary settings. Designing public health measures to control leptospirosis requires an understanding of the ecology of the disease in its many wild and domestic mammalian hosts. The *Leptospira interrogans *complex (sensu lato) includes >200 pathogenic serovars [2], which differ widely in their interactions with different host species. A central tenet of the epidemiology of leptospirosis is the distinction between maintenance hosts and accidental hosts for a given serovar, or, equivalently, between host-adapted and non-host-adapted serovars [1-3]. In this framework, maintenance hosts develop a chronic, largely asymptomatic infection of their proximal renal tubules, and may shed leptospires in their urine for months or years. In contrast, accidental hosts experience acute infections, with symptoms ranging from malaise to multi-organ failure and death.

In California, leptospirosis has resurged in humans and domestic dogs [13,14]. Disease outbreaks have also occurred in California sea lions (*Zalophus californianus*) off the central and northern coasts of California, with hundreds of animals dying in each outbreak. The first leptospirosis epidemic documented in California sea lions was in 1970, and *Leptospira interrogans *serovar Pomona was isolated from infected sea lions [15,16]. Since then there have been repeated epidemics, with an outbreak every three to five years since 1984 [17-19]. The epidemiology of the disease in sea lions is unclear, and the role of sea lions in maintaining this zoonotic disease is unknown. Crucially, it is unknown whether the recurring epidemics of leptospirosis in sea lions result from repeated introduction of the pathogen from external reservoirs, or from cyclic dynamics of an endemic disease. Numerous factors can cause epidemics to cycle, including environmental drivers, changes in host population density, antigenic changes by the pathogen, or changes in the proportion of the host population that is immune, associated with the concept of "herd immunity" [20]. The premise of herd immunity is that an epidemic cannot occur if a certain threshold proportion of a population is immune due to previous infection or vaccination (thus indirectly protecting susceptible individuals against infection). As the pool of susceptible individuals is replenished by birth or immigration, eventually an epidemic becomes possible again.

As the first step to elucidate the epidemiology of recurring leptospirosis outbreaks in California sea lions, and to identify future research directions, this study investigated changes in seroprevalence in an unusually detailed longitudinal dataset from 1995–2005. In an unvaccinated population, seroprevalence is a measure of past exposure to leptospirosis. However, the duration of seropositivity following leptospiral infection is not well known for any host [1,21-23], and is completely unknown for sea lions. Because this is a crucial quantity for interpretation of serological time series, we analyzed available data to characterize the decay rate of antibody titers to leptospires.

## Methods

### Sampling

Serum samples were collected from California sea lions stranding along the central and northern California coast (37°42'N, 123°05'W to 35°59'N, 121°30'W) and archived at The Marine Mammal Center (TMMC) at -70°C. All animals were sampled during routine veterinary care while in rehabilitation as authorized by the National Marine Fisheries Service Research and Enhancement Permit to Take Marine Mammals (# 932-1489-08), and approved by The Marine Mammal Center's Internal Animal Care and Use Committee. For each sex and age class, 20 samples for each year were randomly selected from the archive; when fewer than 20 samples were available for a given sex/age class and year, all samples were used (Table 1). Animals were classified as having stranded because of leptospirosis if serum chemistry results were indicative of leptospirosis (blood urea nitrogen > 100 mg/dl, creatinine > 2 mg/dl, sodium > 155 meq/L and phosphorus > calcium) or if gross necropsy (swollen kidneys with loss of renule differentiation and pale tan renule cortices) and histopathology (interstitial nephritis) revealed renal disease consistent with leptospirosis [19]. To reduce potential bias arising from sampling only stranded sea lions, a reduced dataset was generated which excluded leptospirosis-induced strands.

**Table 1:** Sample sizes for TMMC seroprevalence time series, by sex and age. Total numbers and numbers excluding leptospirosis-induced strands (in parentheses) are shown

**Sex**	**Age**	**1995**	**1996**	**1997**	**1998**	**1999**	**2000**	**2001**	**2002**	**2003**	**2004**	**2005**
Female	Yearling	8	16	19	23	8	18	21	19	38	20	29
	(1–2 y.o.)^a^	(3)	(14)	(17)	(22)	(7)	(11)	(20)	(18)	(38)	(9)	(29)
	Subadult	9	6	19	23	11	19	16	12	20	13	5
	(2–5 y.o.)	(3)	(5)	(13)	(19)	(2)	(13)	(11)	(10)	(16)	(7)	(5)
	Adult	9	7	23	19	18	20	20	18	20	19	20
	(≥ 5 y.o.)	(5)	(5)	(16)	(16)	(13)	(19)	(18)	(17)	(20)	(16)	(19)
Male	Yearling	12	18	19	18	17	17	18	20	38	18	36
	(1–2 y.o.)^a^	(9)	(18)	(15)	(18)	(10)	(12)	(18)	(18)	(38)	(6)	(33)
	Juvenile	21	25	20	20	20	20	19	20	20	19	16
	(2–4 y.o.)	(2)	(16)	(12)	(19)	(3)	(5)	(8)	(9)	(12)	(2)	(11)
	Subadult	19	14	15	20	20	19	13	10	10	19	19
	(4–8 y.o.)	(3)	(10)	(9)	(14)	(4)	(8)	(10)	(6)	(9)	(9)	(9)
	Adult	12	8	9	4	5	8	14	9	8	17	6
	(≥ 8 y.o.)	(7)	(8)	(7)	(3)	(4)	(7)	(12)	(6)	(8)	(9)	(6)

Total		90	94	124	127	99	121	121	108	154	125	131
		(32)	(76)	(89)	(111)	(43)	(75)	(97)	(84)	(141)	(58)	(112)

^a ^The yearling class may include some pups 8–12 months of age, stranding in February to May.

Further serum samples from stranded California sea lions were obtained from two other stranding centers in California, SeaWorld in San Diego, CA (collection range 33°45'N, 118°07'W to 32°32'N, 117°07'W) and the Marine Mammal Care Center at Fort Macarthur, CA (collection range 34°17'N, 119°30'W to 33°45'N, 118°07'W). All available samples were analyzed (Table 2).

**Table 2:** Sample sizes for SeaWorld and Marine Mammal Care Center

**Source**	**Subset**	**2000**	**2001**	**2002**	**2003**	**2004**	**2005**
SeaWorld	Total	4	17	15	58	38	49
	Yearling	0	4	1	17	14	30
MMCC	Total	0	0	0	0	14	30
	Yearling	0	0	0	0	4	12

### Serology

Serum samples were submitted to the California Animal Health and Food Safety Laboratory (Davis, CA), and assayed for Leptospira antibodies using the microscopic agglutination test (MAT) [24-26]. The endpoint reading of the microagglutination reaction was reported as the serum dilution at which 50% of the leptospires were agglutinated by direct observation using inverted field microscopy. Leptospira cultures and serovar-specific control sera were obtained from the USDA National Veterinary Services Laboratories (Ames, Iowa). A previous study evaluated the MAT for sera from California sea lions and reported 100% sensitivity at ≥ 1:3200 based on 19 positive controls (established by clinical signs of disease, lesions at necropsy, and visible leptospires in silver stained kidney sections) and 100% specificity at <1:100 based on 19 negative controls (captive-bred animals that had never exhibited signs of renal disease) [27].

Of the 1344 samples of the 1995–2005 time series, 724 were diluted to a maximum dilution of 1:204800, 17 to a maximum dilution of 1:3200, and 553 to a maximum of 1:800. 50 samples were removed from the analysis due to contamination or incomplete data. The serum samples were assayed against six Leptospira serovars, representing the serovars of diagnostic interest in California during the study period: L. interrogans serovar Pomona, L. interrogans serovar Bratislava, L. kirschneri serovar Grippotyphosa, L. interrogans serogroup Icterohaemorrhagiae serovar Copenhageni, L. interrogans serovar Canicola, and L. interrogans serovar Hardjo type Hardjoprajitno. Most analyses reported here are based on the titer to serovar Pomona, which has been the serovar of all Leptospira isolates from wild California sea lions [15,18,28]. Samples that agglutinated at the 1:100 dilution were classified as seropositive [2,27], and we further distinguished between high-titer seropositives that agglutinated at dilutions of 1:800 or higher [23] and low-titer seropositives that agglutinated only at dilutions below 1:800.

### Analysis

Graphical and statistical analyses were conducted using Matlab v6.1 (The Mathworks, Cambridge MA) and R [29]. Seroprevalences were calculated as binomial proportions with exact confidence intervals [30]. To determine individual risk factors, multinomial logistic regression [31,32] was conducted treating the serologic result (negative, high-titer, low-titer) as a nominal outcome with the following covariates: age, sex, outbreak year (1995,1999,2000,2004) versus non-outbreak year, and season (August-December versus January-July). These seasonal ranges were chosen to minimize the residual deviance of the regression. Outcomes of the multinomial logistic regression are assessed in terms of relative risk ratios (RRR), which describe the ratio between the relative risk of a given outcome versus a reference outcome (e.g. high-titer seropositivity versus seronegativity) for one factor compared to another (e.g. for male versus female individuals). Antibody half-life was estimated as the reciprocal of the slope of the number of two-fold decreases in titer versus time. Throughout the study, proportions were compared using a chi-squared test with continuity correction, or Fisher's exact test when the expected number in any category was <5 [33]. All statistical tests were two-tailed.

Yearling seroprevalence was assessed as an index of leptospirosis exposure by testing for linear relationship with the relative change (i.e. ratio of successive values) in high-titer seroprevalence for the whole population. (The mean of two seroprevalence estimates, derived from the full dataset and from the reduced dataset without leptospirosis strandings, was taken to be the most unbiased estimate of population seroprevalence; qualitatively similar results were obtained using either estimate on its own.) Possible non-linear effects were tested by performing a regression with a quadratic term, and normality of the residuals was assessed using a Kolmogorov-Smirnov test. The intercept of the linear regression, *b*, gives the relative change in high-titer seroprevalence when yearling seroprevalence is zero. If zero yearling seroprevalence indicates no new exposure for the population, then we can calculate the annual per capita probability that antibody decay will cause loss of high-titer status, *p*_*decay*_. Let *N*_*HT *_and *N *be the number of high-titer individuals and total population size, respectively, such that high-titer seroprevalence is *N*_*HT*_/*N*. Let *p*_*death *_be the annual per capita probability of death, and *λ *be the annual growth rate of the population such that *N*(*t*) = *λN*(*t *- 1). If death and antibody decay are independent processes, and there is no new exposure in year *t*, then *N*_*HT *_(*t*) = (1 - *p*_*death*_)(1 - *p*_*decay*_)*N*_*HT *_(*t *- 1). If new recruits to the population are seronegative, as observed for sea lion pups [27], then:

b=NHT(t)/N(t)NHT(t−1)/N(t−1)=[(1−pdeath)(1−pdecay)NHT(t−1)]/[λN(t−1)]NHT(t−1)/N(t−1)=(1−pdeath)(1−pdecay)λ
 MathType@MTEF@5@5@+=feaafiart1ev1aaatCvAUfKttLearuWrP9MDH5MBPbIqV92AaeXatLxBI9gBaebbnrfifHhDYfgasaacPC6xNi=xI8qiVKYPFjYdHaVhbbf9v8qqaqFr0xc9vqFj0dXdbba91qpepeI8k8fiI+fsY=rqGqVepae9pg0db9vqaiVgFr0xfr=xfr=xc9adbaqaaeGacaGaaiaabeqaaeqabiWaaaGcbaqbaeWabmqaaaqaaiabdkgaIjabg2da9KqbaoaalaaabaWaaSGbaeaacqWGobGtdaWgaaqaaiabdIeaijabdsfaubqabaGaeiikaGIaemiDaqNaeiykaKcabaGaemOta4KaeiikaGIaemiDaqNaeiykaKcaaaqaamaalyaabaGaemOta40aaSbaaeaacqWGibascqWGubavaeqaaiabcIcaOiabdsha0jabgkHiTiabigdaXiabcMcaPaqaaiabd6eaojabcIcaOiabdsha0jabgkHiTiabigdaXiabcMcaPaaaaaaakeaajuaGcqGH9aqpdaWcaaqaamaalyaabaWaamWaaeaacqGGOaakcqaIXaqmcqGHsislcqWGWbaCdaWgaaqaaiabdsgaKjabdwgaLjabdggaHjabdsha0jabdIgaObqabaGaeiykaKIaeiikaGIaeGymaeJaeyOeI0IaemiCaa3aaSbaaeaacqWGKbazcqWGLbqzcqWGJbWycqWGHbqycqWG5bqEaeqaaiabcMcaPiabd6eaonaaBaaabaGaemisaGKaemivaqfabeaacqGGOaakcqWG0baDcqGHsislcqaIXaqmcqGGPaqkaiaawUfacaGLDbaaaeaadaWadaqaaGGaciab=T7aSjabd6eaojabcIcaOiabdsha0jabgkHiTiabigdaXiabcMcaPaGaay5waiaaw2faaaaaaeaadaWcgaqaaiabd6eaonaaBaaabaGaemisaGKaemivaqfabeaacqGGOaakcqWG0baDcqGHsislcqaIXaqmcqGGPaqkaeaacqWGobGtcqGGOaakcqWG0baDcqGHsislcqaIXaqmcqGGPaqkaaaaaaGcbaGaeyypa0tcfa4aaSaaaeaacqGGOaakcqaIXaqmcqGHsislcqWGWbaCdaWgaaqaaiabdsgaKjabdwgaLjabdggaHjabdsha0jabdIgaObqabaGaeiykaKIaeiikaGIaeGymaeJaeyOeI0IaemiCaa3aaSbaaeaacqWGKbazcqWGLbqzcqWGJbWycqWGHbqycqWG5bqEaeqaaiabcMcaPaqaaiab=T7aSbaaaaaaaa@A087@

and therefore

*p*_*decay *_= 1 - *bλ*/(1 - *p*_*death*_).

Given approximate values *p*_*death *_= 0.07 and *λ *= 1.05 [34], *p*_*decay *_≈ 1–1.13*b*.

## Results

### Comparison of serovars

To determine the dominant serovar(s) in this dataset, we tabulated which serovar(s) contributed the maximum titer score for each sample with one or more titer ≥ 1:800 (Table 3). Of 449 samples with at least one titer ≥ 1:800, serovar Pomona was the unique maximum for 201 (45%) samples and was positive at the highest dilution measured for an additional 226 (50%) samples. In only 19 of 449 (4%) samples did another serovar have a higher titer than serovar Pomona. Qualitatively similar results were found for all samples with one or more titers ≥ 1:100. Because of the consistently higher titer scores for serovar Pomona, combined with the fact that all *Leptospira *isolates from wild California sea lions have been serovar Pomona [15,18,28], we restrict the remainder of our analysis to serovar Pomona and attribute the positive titers to other serovars to the known cross-reactivity of the MAT [10,35].

**Table 3:** Summary of serovars corresponding to maximum titer scores for all samples with at least one titer ≥ 1:800

**Serovar**	**Pomona**	**Bratislava**	**Canicola**	**Grippo**.	**Hardjo**	**Ictero**.	**Total samples**
Unique maximum^a^	201	9	3	2	2	1	218
Shared maximum (detection limit)^b^	226	212	100	114	79	132	226
Shared maximum (below limit)^c^	3	3	0	2	1	2	5
Titer ≥ 1:800^d^	435	391	221	271	217	302	449

^a ^Samples for which only one serovar had the maximum score.

^b ^Samples for which two or more serovars shared the maximum score, which was the highest dilution measured for that sample.

^c ^Samples for which two or more serovars shared the maximum score, at a titer below the highest dilution measured for that sample.

^d ^Number of samples with titers ≥ 1:800 for each serovar.

### Seroprevalence time series, 1995–2005

**Figure 1:**
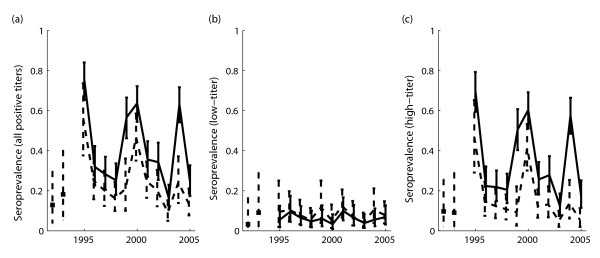
**Cycles in seroprevalence to *L. interrogans *serovar Pomona in California sea lions stranding in northern California**. Three panels correspond to (a) total seroprevalence (titers ≥ 1:100), (b) low-titer seroprevalence (titers ≥ 1:100 and <1:800), and (c) high-titer seroprevalence (titers ≥ 1:800). Solid lines show estimates derived from a random sample of all stranded sea lions, including those judged to have stranded due to leptospirosis. Dashed lines show estimated seroprevalence after leptospirosis strands are removed from the dataset. Isolated points in 1992 and 1993 show estimates derived from non-randomized samples from an earlier publication (*18*). Error bars show 95% confidence intervals.

Seroprevalence of leptospiral antibodies in 1338 stranded California sea lions exhibited cycles of 4–5 year periodicity over the duration of the study (Figure 1a). Peaks in seroprevalence corresponded to reported outbreaks of leptospirosis in 1995, 1999, 2000, and 2004. Cycles were most evident in seroprevalence estimates derived from all stranded sea lions (solid lines); when leptospirosis-induced strands were removed from the analysis (dashed lines), the cyclic pattern remained but the 2004 peak was greatly reduced. The cyclic variation arose in the prevalence of high antibody titers (≥ 1:800) reflective of recent exposure, while the proportion of individuals with lower positive titers was constant within uncertainties (Figure 1b,c).

**Figure 2:**
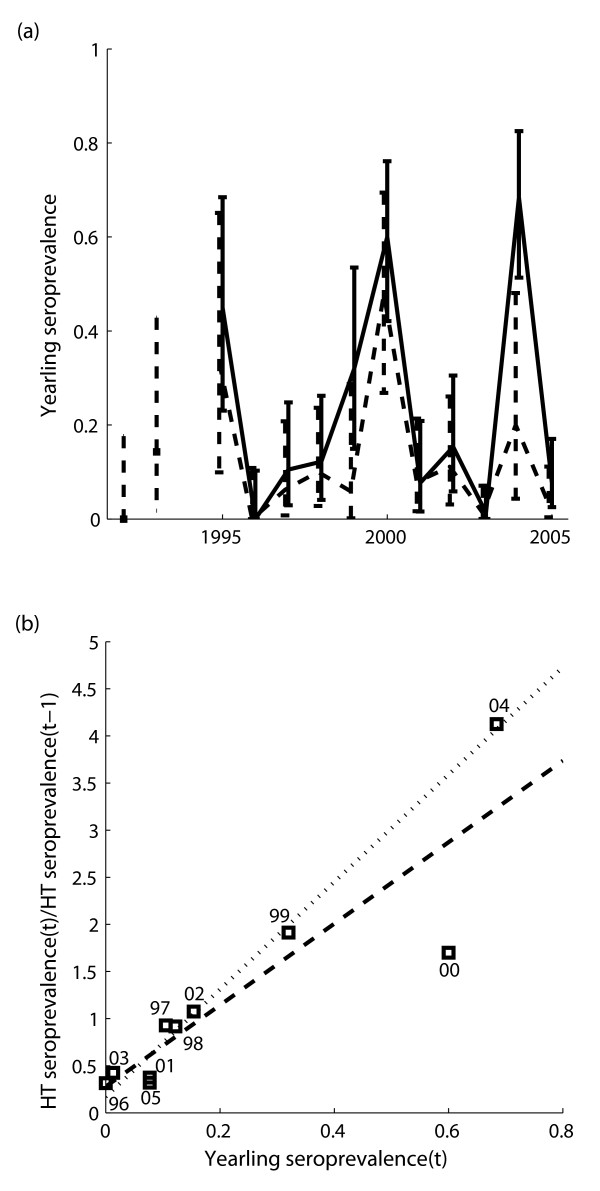
**Yearling seroprevalence and relation to exposure**. (a) Time series of estimated yearling seroprevalence, estimated as in Figure 1. (b) Linear regression of the relative change in high-titer seroprevalence for the whole population against yearling seroprevalence. Squares show data points for particular years (labeled by the final two digits of the calendar year *t*). The dashed line shows the regression including all points; the dotted line shows the regression with the point for the year 2000 excluded.

High-titer seroprevalence remains at 10–30% in non-outbreak years, possibly indicating on-going transmission of leptospirosis. We explored the use of yearling seroprevalence as an index of incidence of leptospirosis in a given year. Sea lion pups remain on the rookery islands for roughly one year after birth [36] and do not appear to be exposed on the rookeries (unpublished data), so yearlings are in their first year of possible exposure to leptospirosis. Yearling seroprevalence exhibited strong peaks in outbreak years, but in intervening years dropped to lower levels than the population seroprevalence (Figure 2a). Seropositive yearlings were observed in all years except 1996 (0/33, 95% CI for binomial proportion: 0–0.11), indicating that exposure to *L. interrogans *serovar Pomona continued at low levels between outbreaks. Yearling seroprevalence was strongly correlated with exposure levels for all ages, as measured by the relative year-to-year change in high-titer seroprevalence (*y *= 0.28 + 4.3*x*, *R*^2 ^= 0.80; Figure 2b). (When the dependent variable was based on seroprevalence estimates with yearlings excluded to avoid possible circularity, the correlation was even stronger (*R*^2 ^= 0.88), but the whole-population results are shown because they inform our work on antibody decay, below.) In 2000, the overall increase in seroprevalence was small compared to a yearling seroprevalence >50%, because of a large number of strandings caused by domoic acid toxicity in that year, which diluted the influence of animals stranding because of leptospirosis [19]. When the point for year 2000 was excluded as an outlier, the linear relationship was much stronger (*y *= 0.17 + 5.7*x*, *R*^2 ^= 0.98; Figure 2b). For both regressions, a quadratic term was not supported and residuals did not deviate significantly from normality.

### Individual risk factors and asymptomatic seropositives

Multinomial logistic regression was performed to assess individual risk factors for high-titer and low-titer seropositivity (Table 4). The relative risk of high titer versus seronegativity was 7.0 times higher in outbreak years than in non-outbreak years, and 4.9 times higher during the August-December season when most leptospirosis strands are reported. The relative risk of high titers was 4.1-fold higher for males than for females. The relative risk of high titers is highest for juveniles and subadults, and drops sharply for adults. Note that the juvenile class contains only males (Table 1), so the sex effect may account for some of the elevated risk for the juvenile class. The relative risk of low titers versus seronegativity was not influenced by outbreak years (*p *= 0.31) or season (*p *= 0.60). In sharp contrast to high-titer results, the relative risk of low positive titers increases steadily with age, with subadults and adults respectively at 24-fold and 44-fold higher risk than yearlings to have low titers.

**Table 4:** Risk factors for high-titer (≥ 1:800) and low-titer (≥ 1:100 and <1:800) seropositivity to *L. interrogans *serovar Pomona. Significant variables in the multinomial logistic regression model and corresponding relative risk ratios (RRR) are shown.^a^

			**High titer seropositivity vs seronegativity**			**Low titer seropositivity vs seronegativity**		
**Variable**	**Value**	**N**^b^	**RRR**	**95% CI**	**p-value**	**RRR**	**95% CI**	**p-value**
Age	Yearling	450 (383)	1			1		
	Juvenile^c^	220 (99)	4.41	(2.80,6.95)	<0.001	5.99	(1.5,23.9)	0.011
	Subadult	331 (195)	3.79	(2.54,5.67)	<0.001	23.8	(6.85,82.6)	<0.001
	Adult	293 (241)	0.79	(0.49,1.27)	0.32	43.6	(12.9,147.4)	<0.001
Sex	Female	565 (456)	1			1		
	Male	729 (462)	4.13	(2.89,5.89)	<0.001	8.29	(4.62,14.9)	<0.001
Season	Jan-Jul	629 (549)	1			1		
	Aug-Dec	665 (369)	4.87	(3.53,6.72)	<0.001	1.15	(0.68,1.92)	0.60
Outbreak year	No	859 (710)	1			1		
	Yes	435 (208)	6.97	(5.02,9.68)	<0.001	1.35	(0.76,2.39)	0.31

^a ^Model had residual deviance 1483.3 on 1287 df (chi-squared *p *= 1; Nagelkerke *R*^2 ^= 0.51).

^b ^Sample size in each group is shown as a total number and as a number excluding leptospirosis-induced strands (in parentheses).

^c ^Note that the juvenile age class contains only males.

Of 514 sea lions with positive titers, 200 stranded for reasons other than leptospirosis; we called these individuals asymptomatic seropositives. The proportion of asymptomatic seropositives was higher among low-titer individuals than high-titer individuals (72/79 versus 128/435, *p *< 0.001). This pattern held for males and females analyzed independently (*p *< 0.001 in each case). The asymptomatic proportion showed a weak tendency to be higher in females than males (*p *= 0.13). A greater proportion of seropositive individuals were asymptomatic in non-outbreak years than outbreak years (*p *< 0.001). The asymptomatic proportion was higher among adults than younger age classes (*p *< 0.001); this pattern held for high titers (*p *< 0.001) but not for low titers (*p *= 0.73). Of the 200 asymptomatic seropositives, 30 stranded due to domoic acid toxicity, 40 due to malnutrition, and the remaining 130 due to varied causes including trauma, cancer, behavioral problems and unrecorded causes; we cannot exclude the possibility that some of these animals stranded due to subclinical leptospirosis.

### Antibody decay and loss of seropositivity

**Figure 3:**
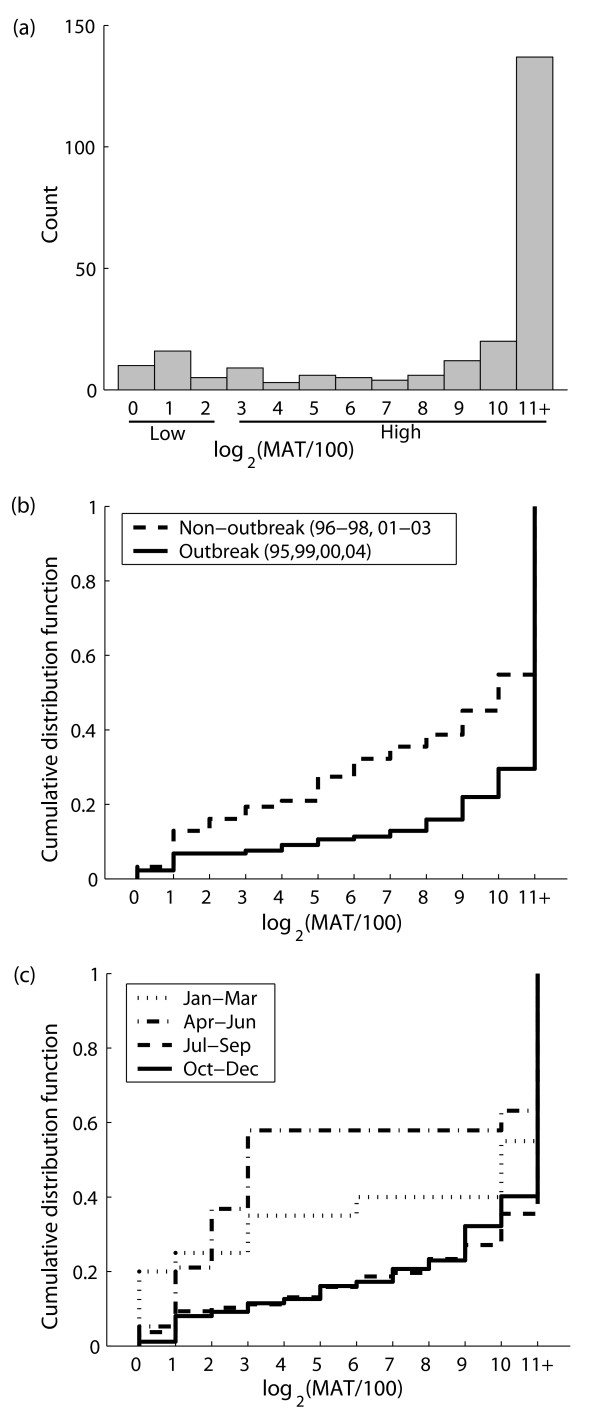
**Distributions of MAT scores from the 1995–2005 time series**. Part (a) shows the histogram of all 248 positive titers among the 724 samples that were measured to a maximum dilution of 1:204800. Parts (b) and (c) show distributions of different subdivisions of these 248 scores, represented as cumulative distribution functions that show the proportion of samples with scores less than or equal to a given value. Titer scores are represented as log_2_(MAT/100), such that a score of 0 corresponds to a titer of 1:100, a score of 1 to a titer of 1:200, etc. The highest score of 11 corresponds to titers ≥ 1:204800. Sample sizes for (c) are Jan-Mar (N = 21), Apr-Jun (N = 21), Jul-Sep (N = 115) and Oct-Dec (N = 91).

Yearling seroprevalence in non-outbreak years was lower than overall seroprevalence. If yearling seroprevalence is an accurate index of transmission within a year (Figure 2b), then this difference indicates that some older individuals maintain seropositivity for one or more years following exposure. The y-intercepts of the regression lines in Figure 2b provide estimates of the proportion of individuals that maintain a high titer from one year to the next without being re-exposed, or conversely the proportion *p*_*decay *_that lose their high titer. When corrections are applied for deaths and recruitment of new unexposed pups (Equation 1), this proportion is estimated to be *p*_*decay *_= 0.69 (95% CI 0.06–1) for the full dataset, or *p*_*decay *_= 0.81 (95% CI 0.59–1) when the 2000 point is excluded. Interpreting these values requires consideration of the quantitative distribution of titer scores. After stimulation by the antigen has ceased, circulating antibodies are conventionally thought to decay exponentially, with half-life of IgG estimated as 23–25 days [37]. Because titers are scored by two-fold dilutions, a drop in titer on a log_2 _scale corresponds to the intervening number of half-lives. Of 248 seropositive samples assayed to dilutions up to 1:204800, 208 were high-titer (≥ 1:800) and 142 were positive at the maximum dilution so their precise titer is unknown (Figure 3a). The remaining 66/208 (32%) of high-titer scores are between 1 and 8 two-fold dilutions from the 1:800 threshold for high titers, so the high titer would be lost after 1–8 half-lives with no re-exposure. However, 142/208 (68%) of high-titer scores are "off the charts", so we cannot assess how many half-lives they are from the threshold. We can conclude only that the titer distributions predict that ≥ 32% of high-titer individuals should drop to low-titer or become seronegative after 8 half-lives without exposure. This is consistent with the regression estimates of 69% and 81%, but does not test the precise values.

Antibody decay is evident in titer distributions during periods of low exposure. Titers from non-outbreak years showed more intermediate values and fewer maximum values than titers from outbreak years (Figure 3b). When stratified by season, titer distributions were heavily skewed toward the highest values during the leptospirosis outbreak seasons of July-September and October-December, then declined progressively to lower values in January-March and April-June (Figure 3c). These patterns cannot be analyzed quantitatively because of seasonal differences in stranding rates across age classes, but the same qualitative patterns arose when yearling or adult samples were excluded from the analysis.

Direct estimates of titer decay rates could be made from six paired serum samples available from California sea lions undergoing rehabilitation following infection. Because the second sample in each instance was seronegative (and we did not know when the detection threshold was crossed), only a minimum bound on decay rate could be estimated from these data. The boundary of the allowable region for decay rates is defined by the best-fit line through the origin and the three fastest-decaying points, which had slope 0.050 (95% CI 0.046,0.054; *R*^2 ^= 0.92) corresponding to a half-life of 20 (19–22) days.

### Comparison with animals stranded in southern California

**Figure 4:**
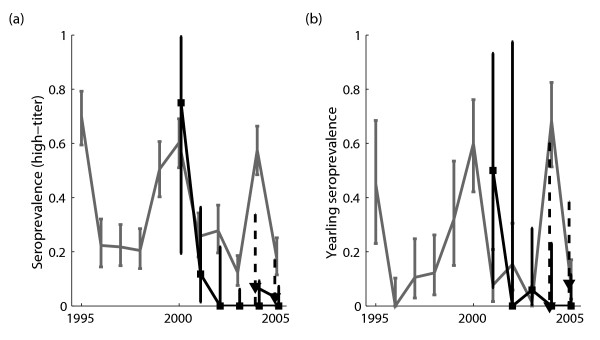
**Seroprevalence in stranded California sea lions: other datasets**. High-titer and yearling seroprevalence from other stranding ranges in southern California. For comparison, estimates from the 1995–2005 time series are shown in gray (full dataset, including leptospirosis-induced strands). Solid lines and squares show estimates from SeaWorld (San Diego CA), while dashed lines and triangles show estimates from the Marine Mammal Care Center (Fort Macarthur CA).

We compared the seroprevalence of sea lions sampled in southern California to the TMMC time series (Figure 4). The 2000 outbreak was reflected in the high-titer seroprevalence from SeaWorld (though sample size was only *n *= 4). The 2004 outbreak was not evident at either southern California site: high-titer seroprevalence was greater at TMMC than MMCC (*p *= 0.002) and SeaWorld (*p *< 0.001), as was yearling seroprevalence (*p *= 0.047 and *p *< 0.001, respectively).

## Discussion

Leptospirosis in California sea lions does not fit neatly into the classical dichotomous framework wherein a given host species is either a maintenance host or an accidental host for a particular serovar of pathogenic *Leptospira*. In sea lions the disease exhibits characteristics of accidental hosts, with pathogenic and sometimes fatal outcomes for individual animals and dramatic outbreaks at the population scale. Yet the disease also appears to circulate at low levels between outbreaks, and asymptomatic seropositivity (even with high titers) is common among adults. Other investigators have reported chronic shedding of leptospires, another characteristic of classical maintenance hosts, with one sea lion reported to shed for at least 154 days following infection [17].

All available evidence, including several isolates from wild sea lions [15,18,28] and comparison of MAT titer scores (Table 3), points to *L. interrogans *serovar Pomona as the cause of leptospirosis in this population. Mixing of host-adapted and non-adapted traits may be a property of serovar Pomona, which causes disease in pigs, cattle and horses, but can also be shed for 4–6 months by those species [38-40]. Of course, the possibility that other serovars are circulating cannot be excluded without intensive efforts to isolate and identify further leptospires from wild sea lions. Two serovars that were not included in our MAT panel warrant special mention.*L. interrogans s*erovar Autumnalis is increasingly reported in serological studies of dogs in the United States [11] and is known to cross-react with Pomona in the MAT [11,41]. *L. kirschneri *serovar Cynopteri has been detected serologically in a geographically separated population of *Z. californianus *in the Gulf of California [42], but the highest titer observed (1:50) was below our threshold for seropositivity. On-going circulation of serovar Pomona remains the most parsimonious explanation for the available data.

If the classical maintenance/accidental-host model of leptospirosis epidemiology is overly simplified, then public health officials must broaden their view of potential reservoirs for this zoonotic pathogen. If the California sea lion population is indeed a reservoir for serovar Pomona, then health warnings regarding leptospirosis risk from stranded sea lions should be extended to non-outbreak periods. The possibility that on-going exposure of sea lions to serovar Pomona arises from continuous contact with an unidentified external reservoir cannot be excluded, but this explanation would only broaden the public health implication to include other host species.

The present study identifies clear cycles of 4–5 year periodicity in seroprevalence to *L. interrogans *serovar Pomona in the California sea lion population off the California coast. The cyclic pattern arises from changes in the prevalence of high titer scores reflective of recent exposure, and peak years of the cycle correspond to observed peaks in sea lions stranding with leptospirosis. Seroprevalence in yearling sea lions is strongly correlated with annual changes in high-titer seroprevalence for the whole population, and indicates on-going exposure to serovar Pomona between outbreak years; this finding is consistent with earlier studies reporting continued stranding and death due to leptospirosis in non-outbreak years [18,19], and inconsistent with expected patterns for an accidental host. Evaluation of individual risk factors reveals that juvenile and subadult animals are at greatest risk for high-titer seropositivity, while adults are at sharply reduced risk.

These observations suggest strongly that leptospirosis is endemic within the sea lion population, and raise the intriguing possibility that repeated epidemics arise from the intrinsic interaction of birth rates and herd immunity, rather than the environmental drivers that are commonly postulated. Younger animals get infected in outbreak years, acquiring high titers that may persist for a year or more, while most adults are immune from previous exposure. The pathogen may persist through off-seasons and non-outbreak years via chronic infections and a low level of on-going transmission, possibly associated with the increased prevalence of asymptomatic seropositivity and low titers during these periods. We emphasize, though, that present data cannot exclude the possibility of on-going contact with another reservoir species. Mathematical models integrating the dynamics of disease transmission and immunity with sea lion demographics, combined with further data collection, are essential to clarify this issue.

Live-stranded marine mammals are a biased sample of the wild population, over-representing sick and weak individuals, so seroprevalence in stranded individuals may exceed the true population value. We addressed this bias by providing alternate seroprevalence estimates based on a reduced dataset excluding clinical leptospirosis cases, but this approach may yield underestimates during outbreaks if a substantial proportion of infected animals do not come ashore. Of over 200,000 sea lions breeding off the California coast [34], just a few hundred leptospirosis cases strand and are admitted to rehabilitation during a typical outbreak year [19]. Given peak seroprevalence estimates >50%, it appears that many infected animals do not strand. Serosurveillance of free-ranging sea lions is crucial to determine how the patterns reported here scale to the population level.

Data from sea lions stranded in southern California showed a general trend of lower seroprevalence than was found in central and northern California, and, intriguingly, there is no evidence of the 2004 outbreak in data from the southern range. All California sea lions in the eastern Pacific Ocean breed on rookery islands off the coast of southern California and the Baja peninsula, so animals stranding in different regions of California are thought to be drawn from a single population. Leptospirosis strandings (and high-titer seropositivity) peak during July to November [19], when sea lions migrate northward following the breeding season to forage off central and northern California, or points further north. It is unknown whether this timing is coincidental or leptospirosis transmission (or exposure) is aided by environmental factors in the northern range. Male sea lions migrate further north than females, and in greater numbers, while breeding females remain closer to the rookeries to nurse pups. This difference in migratory behavior may play a role in the observed sex difference in leptospirosis incidence, although similar sex differences have been observed in other species including humans and are not easily explained [13,43]. Data presented here suggest that leptospirosis incidence is lower among animals remaining in southern California, but increased sampling of stranded and wild-caught individuals is needed to confirm this pattern.

The predominance of high titers among seropositive sea lions (also reported by Colagross-Schouten [27]) indicates that most animals had been exposed recently before stranding. Yet the time course of titer decay for this host-serovar interaction is unknown, so we assembled evidence to assess the duration of high MAT titers in sea lions. Population-level changes in seroprevalence suggest that approximately 69% of high-titer individuals lose their high titer in a year without re-exposure to the pathogen (but note the broad 95% CI, 0.06–100%). This is consistent with observed titer distributions in sea lions, which indicate that ≥ 32% of individuals should lose their high titers after 8 antibody half-lives, but more precise predictions cannot be derived from titer distributions because the majority of sea lion titers were positive at the highest dilution measured. Maintenance of high MAT titers for several years is not reported in experimental infection studies, which rarely last that long, but has been reported for humans following severe infections [1,21-23]. MAT titer half-life for sea lions in rehabilitation was estimated crudely to have upper bound 20 (19–22) days, lower than the conventional half-life of 23–25 days for IgG antibodies. While this difference could be attributed to numerous factors, including that MAT titers reflect both IgM and IgG levels [1] and antibiotic therapy may reduce titer duration [35], it is important to note the small sample size underlying the estimate, and the well-established finding that human MAT decay rates vary substantially [21-23]. Titer distributions in stranded sea lions are qualitatively consistent with gradual antibody decay following exposure, but intermediate titers (from 1:3200 to 1:51200) appear less commonly than a simple exponential decay model would predict. Longitudinal titers from individuals recovering from acute infection are required to characterize the true rate of antibody decay from high levels. The possible role of chronic shedders, in maintaining their own low-titer seropositivity and in boosting the antibody responses of others, requires investigation.

## Conclusion

This study presents results of an unprecedented serosurveillance program in marine mammals, tracking seroprevalence to *L. interrogans *serovar Pomona in 1344 stranded California sea lions over 11 years. The data show cycles of 4–5 year periodicity, with peaks corresponding to observed increases in sea lion strandings due to leptospirosis. Seroprevalence in yearling sea lions is an accurate indicator of incidence of leptospiral infection for all age classes, and indicates on-going exposure to serovar Pomona between outbreak years. These results suggest that sea lions occupy a middle ground between classically-defined maintenance and accidental hosts of leptospirosis, but many questions remain. Further data are needed to address crucial uncertainties such as the seroprevalence of the free-ranging population, the prevalence of chronic shedding among sea lions, and the spatiotemporal interaction of sea lion migration and leptospirosis risk. Molecular comparisons of *L. interrogans *isolates from sea lions and possible external reservoirs would contribute vital evidence regarding the question of endemic persistence versus repeated introductions. Further analysis of existing data is also essential, particularly via mathematical models that integrate the dynamics of disease transmission, population growth, and antibody decay with all available data types. The sea lion/leptospirosis system raises questions regarding the accepted view of the epidemiology of this important zoonosis, but only through a sustained interdisciplinary effort will definitive answers be obtained.

## Competing interests

The author(s) declare that they have no competing interests.

## Authors' contributions

JL-S analyzed the data and drafted the manuscript. DG managed the samples and primary data at TMMC, and helped to interpret the results and draft the manuscript. GG coordinated the early phase of the data collection. SH coordinated the serological analysis and advised on its interpretation. LP and JSL contributed samples and accompanying data from southern California. BG advised on the analysis and interpretation of the time series, and helped to draft the manuscript. FG conceived of the study, participated in its design and coordination, and helped to interpret the results and draft the manuscript. All authors read and approved the final manuscript.

## Pre-publication history

The pre-publication history for this paper can be accessed here:


